# Time-restricted Eating to Address Cancer-related Fatigue among Cancer Survivors: A Single-arm Pilot Study

**Published:** 2022-05-30

**Authors:** Amber S. Kleckner, Brian J. Altman, Jennifer E. Reschke, Ian R. Kleckner, Eva Culakova, Richard F. Dunne, Karen M. Mustian, Luke J. Peppone

**Affiliations:** 1 Department of Pain and Translational Symptom Science, University of Maryland School of Nursing, Baltimore, MD, USA; 2 Greenebaum Comprehensive Cancer Center, Baltimore, MD, USA; 3 Department of Biomedical Genetics, University of Rochester Medical Center, Rochester, NY, USA; 4 Wilmot Cancer Institute, Rochester, NY, USA; 5 Division of Supportive Care in Cancer, Department of Surgery, University of Rochester Medical Center, Rochester, NY, USA; 6 Department of Medicine, University of Rochester Medical Center, Rochester, NY, USA

**Keywords:** Oncology, Fatigue, Intermittent fasting, Nutrition, Diet, Supportive care, Behavior

## Abstract

**Purpose:**

Cancer-related fatigue is a prevalent, debilitating condition that can persist for months or years after treatment. In a single-arm clinical trial, the feasibility and safety of a time-restricted eating (TRE) intervention were evaluated among cancer survivors, and initial estimates of within-person change in cancer-related fatigue were obtained.

**Methods:**

Participants were 4–60 months post-cancer treatment, were experiencing fatigue (≥ 3 on a scale 0–10), and were not following TRE. TRE entailed limiting all food and beverages to a self-selected 10-h window for 14 days. Participants reported their eating window in a daily diary and completed the Functional Assessment of Chronic Illness Therapy-Fatigue (FACIT-F), Brief Fatigue Inventory (BFI), and symptom inventory pre- and post-intervention. This study was pre-registered at clinicaltrials.gov in January 2020 (NCT04243512).

**Results:**

Participants (n=39) were 61.5 ± 12.4 years old and 1.8 ± 1.3 years post-treatment; 89.7% had had breast cancer. The intervention was feasible in that 36/39 (92.3%) of participants completed all questionnaires and daily diaries. It was also safe with no severe adverse events or rapid weight loss (average loss of 1.1 ± 2.3 pounds, *p*=0.008). Most adhered to TRE; 86.1% ate within a 10-h window at least 80% of the days, and the average eating window was 9.33 ± 1.05 h. Fatigue scores improved 5.3 ± 8.1 points on the FACIT-F fatigue subscale (*p*<0.001, effect size [ES]=0.55), 30.6 ± 35.9 points for the FACIT-F total score (*p*<0.001, ES=0.50), and −1.0 ± 1.7 points on the BFI (*p*<0.001, ES=−0.58).

**Conclusion:**

A 10-h TRE intervention was feasible and safe among survivors, and fatigue improved with a moderate effect size after two weeks.

**Limitations:**

This was a single-arm study, so it is possible that expectation effects were present for fatigue outcomes, independent of effects of TRE *per se*. However, this feasibility trial supports evaluation of TRE in randomized controlled trials to address persistent cancer-related fatigue.

## Introduction

Cancer-related fatigue affects at least 30–90% of patients who undergo treatment for cancer treatment, including both chemotherapy and radiation [1]. It is one of the most prevalent and debilitating side effects of these treatments, and can persist for years into survivorship [2,3]. Cancer-related fatigue is tiredness that is not relieved by sleep or rest, and it contributes to substantial adverse physical, psychological, and economic consequences as well as increased mortality [2,4]. Development of effective treatments has been hindered by the lack of knowledge of the etiology and pathophysiology of cancer-related fatigue [5].

“Time-restricted eating” refers to restricting eating to a consistent time window between 6 and 12 hours without an overt attempt to reduce caloric intake. Time-restricted eating as a therapeutic approach has garnered substantial appreciation in the literature and among the public in the last decade for its effectiveness to sustain circadian rhythms to prevent and treat disease [6]. Human and rodent studies have shown that time-restricted eating prevents excess weight gain, improves sleep, and slows age- and diet-induced disease [6]. Until recently, time-restricted eating had been studied only in healthy participants or those who are overweight or with metabolic disorders (e.g., [7–13]). These studies collectively show that a 10-hour time-restricted eating window is feasible, safe, and effective at improving metabolic markers [7–11]. Two studies looked at fatigue as a potential consequence of time-restricted eating—in a study among 11 men who were overweight or obese, five days of time-restricted eating in an 8-hour window did not affect fatigue levels [11]. Also, among eight overweight adults, 16 weeks of time-restricted eating in a 10–12-hour window led to energy levels were significantly greater overall and in the mornings specifically, and improvements in energy were sustained at one year [14]. However, there have not yet been published studies, to our knowledge, in the cancer population [8].

Thus, we hypothesize that time-restricted eating can help alleviate persistent cancer-related fatigue. Herein, we performed a two-week, single-arm pilot study that evaluated the feasibility of recruiting cancer survivors to a 10-hour time-restricted eating study, adherence of the participants to the program, and safety of a 10-hour time-restricted eating program in regard to adverse events with a special attention to weight loss. Further, we assessed within-person changes in patient-reported fatigue from pre- to post-intervention.

## Methods

### Study design, participants, and procedures

This prospective single-arm pilot clinical trial was conducted at University of Rochester Medical Center (URMC) from June 2020-September 2021. This study was pre-registered at clinicaltrials.gov on 28/01/2020 (NCT04243512). The research protocol was reviewed and approved by the Research Subjects Review Board at URMC (study no. 00004598). All methods were performed in accordance with the Declarations of Helsinki.

Participants were eligible if they had completed adjuvant chemotherapy, surgery, and/or radiation for cancer at least 4 months and not more than 5 years prior to enrolling; had a baseline level of fatigue as determined by reporting a score of 3 or greater for the question, “In the last week, how bad was your worst fatigue on a scale from 0–10?”; spoke English; were at least 18 years old; were not already in the habit of eating all their food within a 10–hour window; had a body mass index (BMI) >20.0 kg/m^2^; did not have surgery planned in the next month; did not have any contraindications to the proposed nutrition intervention as identified by their medical provider, their designee, or the study team (e.g., high risk for hypoglycemia, medication requirements, recent history of an eating disorder); were not taking insulin; and were not taking enteral or parenteral nutrition.

While all participants were recruited locally, all recruitment and study activities were accomplished remotely during the coronavirus disease 2019 (COVID-19) pandemic. Participants were provided the choice to consent via a paper consent (via mail) or eConsent using REDCap software [15,16]. Participants completed baseline procedures before making any changes to their diet patterns, i.e., questionnaires, 24-hour food record, and body weight. They then were asked to follow time-restricted eating for 14 consecutive days. At the mid-point, a nutritionist on the study team called the participant to check in, inquire about adverse events, and discuss barriers to time-restricted eating. Post-intervention data collection (i.e., questionnaires, body weight, 24-hour food record) occurred on Day 14. Within a week of completing the intervention, study staff conducted a semi-structured interview to get feedback on participants’ experience with the intervention.

### Time-restricted eating intervention

The restricted eating window was 10 hours long, during the day, and was selected by the participant based on their normal meal patterns and preferences. We asked that the window be consistent during the study period. Water and medications were allowable any time but, because of the potential of caffeine and artificial sweeteners to affect circadian rhythm [17,18], coffee, tea, chewing gum, and diet beverages were discouraged during the fasting window.

### Data collection

Participants completed a paper-based daily diary for each of the 14 days of the intervention, which included the time of their first calorie and time of their last calorie. The daily eating window was calculated as the difference between first and last calorie. The *a priori* primary outcome was adherence to time-restricted eating, as calculated from the percent of days that each participant adhered to the intervention.

At baseline and Day 14, a battery of three questionnaires was administered to assess fatigue and other symptoms-the Functional Assessment of Chronic Illness Therapy-Fatigue (FACIT-F) [19], Brief Fatigue Inventory (BFI) [20], and a Symptom Inventory. The FACIT-F is a validated, common measure of fatigue that is comprised of five subscales: physical well-being, social well-being, emotional well-being, functional well-being, and fatigue [19]. It asks how true 40 statements are over the last 7 days such as “I have lack of energy” and “I have trouble starting things because I am tired” with five response choices ranging from 0, “Not at all,” to 4, “Very much.” The BFI is a 10-item fatigue questionnaire that is also validated and commonly used in the cancer population [20]. It captures fatigue *now* as well as the *usual* and *worst* in the last 24 hours. It also includes six single-item questions regarding how fatigue has interfered with general activity, mood, work, etc. The average of all 10 items yields a global fatigue score. Cronbach alpha reliability ranges from 0.82 to 0.97 [21]. Lastly, the Symptom Inventory consisted of 29-items that captured 21 symptoms and 8 questions regarding how many their symptoms interfered with daily activities, mood, and relationships. We report data on fatigue, sleep problems, drowsiness, and interference of symptoms with quality of life.

A 24-hour food record was collected at baseline and Day 14, in which participants were asked to record all food and beverage intake, including time eaten and portion size. These data were entered into NDSR software (Nutrition Coordinating Center, University of Minnesota, Minneapolis, MN) and analyzed for total caloric intake.

Adverse events were closely monitored as described by the Common Terminology Criteria for Adverse Events (CTCAE), version 5 [21], with special attention to body weight. Each participant was provided a bathroom scale (Weight Watchers by Conair, Stamford, CT). Participants were instructed to place the scale on a hard, level surface and weigh themselves shortly after rising the mornings of Day 1, Day 8, and Day 15. Body weight was recorded by the participant on their daily diary.

### Statistics

Descriptive analyses (count, mean, standard deviation [SD], range, percentage, as appropriate) were used to describe the study sample demographics, feasibility metrics, and outcomes. A paired t-test was used to assess symptom measures at post- vs. pre-intervention for participants with evaluable data at both time points. Effect size (ES) was calculated as the change in the measure from pre- to post-intervention divided by the pooled SD. *p*<0.05 was deemed statistically significant.

## Results

**Figure 1.**
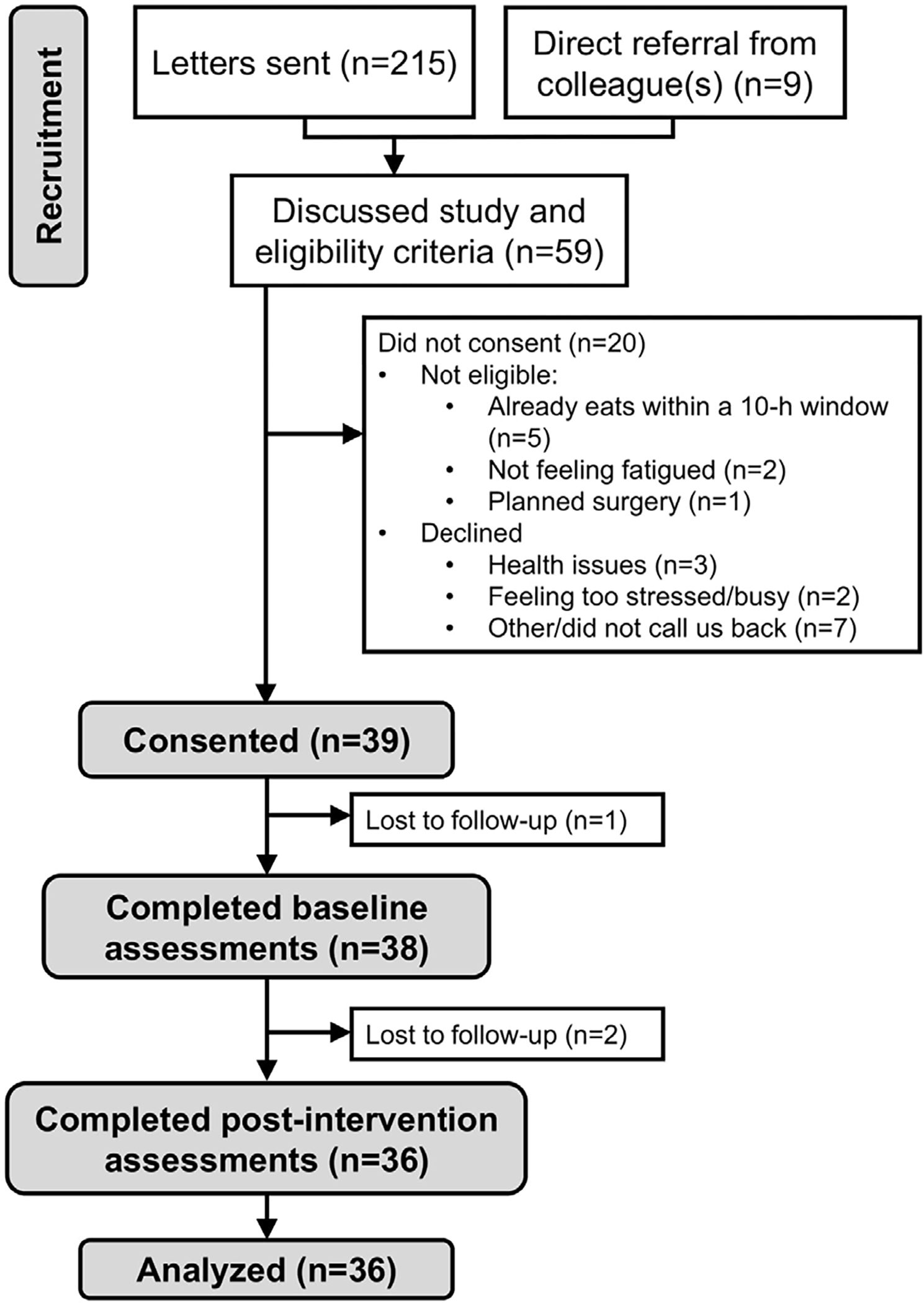
CONSORT flow diagram.

A total of 215 letters were sent to potential participants and we had nine referrals from colleagues. We then discussed our study with 59 potential participants (Figure 1). Of these 59, 39 were eligible and volunteered to participate (66.1% recruitment rate). A total of 36 participants provided data at baseline and Day 14 for a retention rate of 92.3%.

Participants were 61.5 ± 12.4 years old, 92% female, and 84.8% were White, non-Hispanic (Table 1). The majority (89.7%) of participants had been treated for breast cancer, and the majority (71.8%) had had stage 0 or 1 cancer. Among the 36 women, 25 (69.4%) were post-menopausal. Eight (20.5%) participants were normal weight (BMI <25 kg/m^2^), eight (20.5%) were overweight (25 ≤ BMI <30 kg/m^2^), and 23 (59.0%) were obese (BMI ≥ 30 kg/m^2^).

Our primary aim was to assess adherence of the participants to the 10-h time-restricted eating intervention. Daily diaries were collected from 36 participants (92.3%), who completed 100% of entries. On average, participants adhered to the 10-h window 90.1% of the days, and the average eating window was 9 hours, 20 minutes (± 1 hour, 3 minutes). A total of 86.1% of participants adhered more than 80% of the days, which was larger than our *a priori* minimum of 80% to declare the program feasible. In regard to safety, there were two Grade 1 adverse events—one instance of headache, which was possibly related to the intervention, and one instance of insomnia, which was unlikely to be related to the intervention. Both occurred within the first few days of starting time-restricted eating. There were no other adverse events, including severe adverse events. Participants lost 1.1 ± 2.3 pounds over the course of the study (*p*=0.008). Based on 24-hour food logs, participants (*n*=35) consumed 1858 ± 554 kcal at baseline and 1663 ± 462 kcal on Day 14, a difference of 202 ± 654 kcal (10.5 ± 35.2%) fewer total calories (*p*=0.076).

**Figure 2.**
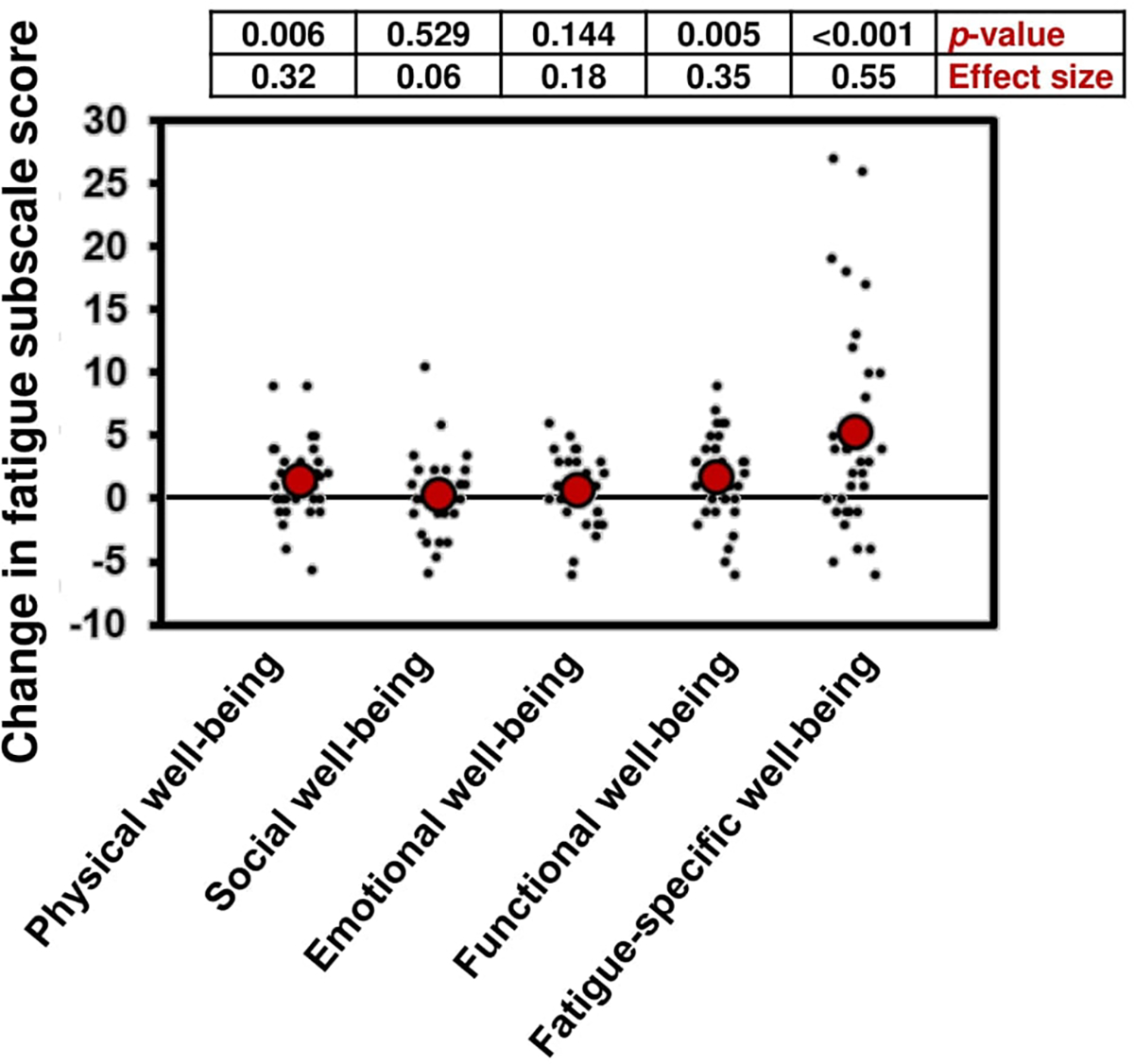
Change in subscales of the Functional Assessment of Chronic Illness Therapy-Fatigue (FACIT-F) from baseline to Day 14 while participating in a 10-hour time-restricted eating regimen. A greater score indicates greater quality of life and less fatigue (n=36).

Fatigue was measured at baseline and post-intervention using the multidimensional FACIT-F, the BFI, and the symptom inventory (Table 2). Total FACIT-F total score changed by 9.3 ± 13.3 (mean ± SD) points indicating improvements in fatigue (*p*<0.001, ES=0.50). For the FACIT-F fatigue subscale, fatigue improved by 5.3 ± 8.1 points (*p*<0.001, ES=0.55, Figure 2), which is greater than the minimal clinically important difference (MCID) of 3 points [22]. Fatigue also improved 8.4 ± 11.2 points according to the FACIT-F Trial Outcome Index, which is more than the MCID of 5.0 points [22]. Significant improvements were also observed for change in physical well-being (1.4 ± 2.9 points), functional well-being (1.7 ± 3.3 points), and quality of life (as measured from the Functional Assessment of Cancer Therapy-General [FACT-G] subscale, 4.0 ± 7.0 points, *p*<0.01 for all). The BFI reflected improvements in fatigue, with global scores changing −1.0 ± 1.7 points (*p*<0.001, ES= −0.58, Table 2). Single-item questions on the symptom inventory regarding fatigue and drowsiness reflected improvements as well as how symptoms interfered with quality of life, but there was not a significant change in reported sleep problems (Table 2).

Exit interviews were conducted within a week of completing the study (n=37). Overall, participants enjoyed time-restricted eating and many indicated that it facilitated a more regular eating pattern. Several people reported that it took about three days to one week for their bodies to adjust to the new pattern, after which it was easier and more comfortable. The vast majority voiced that they would continue time-restricted eating in some capacity, some with modifications such as drinking coffee earlier. Some did not like the strictness of the time window but said that they benefited from not eating after dinner and will continue to follow that practice. All participants would recommend time-restricted eating to friends or family, often with specific goals including weight management/loss, regulating blood sugar, reducing fatigue, or promoting overall health. The largest barriers to time-restricted eating included coordinating with their work schedule, coordinating meal times with their family/spouse, and not being allowed to drink caffeinated beverages in the morning. Through this study, some participants reflected on other lifestyle behaviors including diet (i.e., composition and quantity of food) and physical activity.

## Discussion

Herein, we demonstrated feasibility of a 14-day, 10-hour time-restricted eating intervention among cancer survivors with persistent cancer-related fatigue, as well as initial efficacy. The intervention was very well received, and the vast majority of participants stated that they will continue a time-restricted eating in some fashion. This intervention was safe, and participants reported clinically meaningful improvements in their fatigue using uni- and multidimensional fatigue questionnaires. These results support further testing of a 10-hour time-restricted eating intervention vs. usual care and time- and attention-control interventions to alleviate cancer-related fatigue in cancer survivorship.

Adherence was excellent, with 86% adhering at least 80% of the days, and an average eating window of 9 hours, 20 minutes. This is in line with other studies that have evaluated adherence to 8–10-hour time-restricted eating regimens. For example, healthy participants adhered to a 10–12-hour window (self-selected) 6.5 ± 0.5 days/week during a 12-week-long study (92% adherence, n=19) [7]. Adults with type 2 diabetes adhered to a 9-hour eating window (10:00–17:00) 20 ± 7 out of 28 days of the intervention (average 72% adherence, n=19) [10]. Obese adults adhered to an 8-hour eating window (10:00–18:00) 5.6 ± 0.3 days/week for 12 weeks (80% adherence, n=23) [23]. Further, people with metabolic syndrome adhered at least 5.0 ± 2.2 days/week over a 12-week intervention (72–81% weekly adherence, n=37). While our study was only 14 days long, other studies have demonstrated consistent adherence over time periods up to 12 weeks [23,24].

Our results build upon literature showing that cancer survivors with persistent cancer-related fatigue are willing and able to adhere to behavioral interventions to address their fatigue. Currently, behavioral interventions such as exercise and psychosocial interventions (e.g., cognitive behavioral therapy) are more effective to combat fatigue than the available pharmaceuticals [25], and effective nutritional interventions are emerging [26]. Behavioral interventions are desirable because they have a plethora of health benefits and few side effects. Time-restricted eating, specifically, is appealing because it is free-of-charge and does not require specialized equipment, thereby making it widely accessible [8]. Our adherence rate of 90% was slightly higher than many other studies that evaluate behavioral interventions among cancer survivors. For example, exercise interventions tend to have adherence rates of 54–78% among cancer survivors [27]. Dietary interventions tend to have more variable adherence, with rates ranging from less than 50% to 100%, depending on how adherence is calculated [26]. Notable examples include adherence of 81% at 12 weeks for a Mediterranean diet intervention [28] and 73–94% for food group goals in a 12-week ‘Fatigue Reduction Die’ study [29], both of which were conducted among survivors with persistent cancer-related fatigue.

Time-restricted eating has the potential to improve several pathophysiological mechanisms that underlie cancer-related fatigue. First, time-restricted eating may be able to alleviate fatigue by entraining circadian rhythms. Circadian rhythms are 24-hour biological cycles that work in synchrony to regulate hormone secretion, the sleep/wake cycle, and metabolic processes. Cancer and cancer treatment have been shown to disrupt circadian rhythm, which can lead to sleep disturbances and fatigue [30–33]. Circadian rhythm is affected by external cues—zeitgebers—that include light exposure, sleep, physical activity, and nutrient timing [6]. Consistent animal and human data demonstrate that aberrant eating patterns disrupt circadian rhythm (i.e., disruption of expression of genes that show strong diurnal oscillations) [6]. Only about 10% of people eat within a window less than 12 hours [14], and a consistent, shorter window of eating, for example 10 hours, may help entrain the circadian clock and improve metabolic homeostasis with broad health consequences [6,7]. In addition, bright light therapy, which aims to regulate circadian processes, has shown benefits for cancer-related fatigue [34]. Second, cancer and cancer treatments can interfere with metabolism including glucose, lipid, and redox homeostasis, hormone regulation, and mitochondrial function, possibly causing or exacerbating cancer-related fatigue [5,35,36]. Time-restricted eating can regulate glucose metabolism and metabolic hormones in people with chronic conditions [6,7,24]. It can be combined with other dietary interventions that prescribe the amount and/or composition of the diet (e.g., [9]). Other suggested mechanisms underlying cancer-related fatigue include chronic inflammation, disruption of the hypothalamus-pituitary-adrenal (HPA) axis, neuroendocrine functions, and psychological distress [5,36]. There is preliminary evidence that time-restricted eating can regulate and/or improve some of these functions via circadian or independent processes [8,37]. The effects of time-restricted eating on circadian rhythm have yet to be measured and the relationships between cancer- and treatment-related pathology, persistent psychological distress, circadian rhythm, and cancer-related fatigue are not yet fully elucidated, but this is a rich area for future research.

The results of this study should take into account both its strengths and limitations. It is one of the first studies testing time-restricted eating in the cancer population, and the first, to our knowledge, to apply it to address chronic fatigue and supportive care outcomes. Our study is limited in that it was small and conducted in mostly older breast cancer survivors, so generalizability should occur with prudence. Also, recruitment occurred during 2020–2021; it is unknown how the COVID-19 pandemic impacted the feasibility of recruitment and participants’ ability to adhere to time-restricted eating. However, because our study was conducted completely remotely, our methods may be used for follow-up multisite studies and studies in rural and other hard-to-reach populations. Lastly, our study was only 14 days long and time-restricted eating interventions that report health benefits are usually at least 8 weeks [6,8]; it is unknown how adherence will change over a longer study period.

## Conclusion

Cancer survivors with persistent cancer-related fatigue were willing and able to adhere to a 10-hour time-restricted eating intervention for two weeks with eagerness to continue the eating pattern. Time-restricted eating was safe and fatigue improved significantly from pre- to post-intervention with moderate effect sizes. These results support future work investigating the effects of time-restricted eating against usual care and other behavioral control interventions to address cancer-related fatigue.

## Figures and Tables

**Table 1.** Demographics and clinical characteristics (n=39*).

Characteristics	Mean ± SD or n (%)
Age (years)	61.5 ± 12.5
Gender	
Male	3 (7.7%)
Female	36 (92.3%)
Race and ethnicity	
African American/Black	4 (10.3%)
Asian	1 (2.6%)
Hispanic/Latinx	1 (2.6%)
White, non-Hispanic	33 (84.6%)
Marital status	
Married or long-term committed significant other	28 (71.8%)
Divorced, separated, single, or widowed	10 (25.6%)
Employment	
Employed (including self-employed)	18 (46.2%)
Home Maker	6 (15.4%)
Unemployed	12 (30.8%)
Highest level of education	
High school/GED or less	19 (71.8%)
2 or 4 year degree or some college	17 (43.6%)
Graduate degree	2 (5.1%)
Body mass index (kg/m^2^)	32.3±7.1
Type of cancer	
Breast	35 (89.7%)
Prostate	3 (7.7%)
Uterine	1 (2.6%)
Cancer stage	
0	5
1	23
2	7
3 or 4	3
Previous treatment for cancer	
Surgery	36 (92.3%)
Chemotherapy	14 (35.9%)
Radiation	35 (89.7%)
Years since treatment	1.7±1.2

* One participant did not complete the On Study form at baseline. n=38 for marital status, employment, and education

**Table 2.** Fatigue measured at baseline and after 14 days of following a 10-hour time-restricted eating pattern (n=36).

Fatigue measure	Directionality	Baseline	Day 14	*p*-value*	Effect size
Functional Assessment of Chronic Illness Therapy- Fatigue (FACIT-F) Total score	Higher is better	107.9 ± 17.3	117.2 ± 19.6	<0.001**	0.50
FACIT-F: Physical well being	Higher is better	19.9 ± 4.1	21.4 ± 4.7	0.006**	0.32
FACIT-F: Social well being	Higher is better	22.8 ± 5.7	23.1 ± 5.5	0.529	0.06
FACIT-F: Emotional well being	Higher is better	18.5 ± 3.7	19.1 ± 3.5	0.144	0.18
FACIT-F: Functional well being	Higher is better	16.0 ± 4.5	17.3 ± 4.9	0.005**	0.35
FACIT-F: Fatigue subscale	Higher is better	30.6 ± 9.2	35.9 ± 9.9	<0.001**	0.55
FACIT-F: Trial outcome index (fatigue)^†^	Higher is better	66.6 ± 14.8	74.9 ± 17.3	<0.001**	0.52
FACIT-F: Functional Assessment of Cancer Therapy (FACT)-General^‡^	Higher is better	77.2 ± 12.3	81.3 ± 13.0	0.001**	0.32
Brief Fatigue Inventory: Global fatigue score	Lower is better	3.9 ± 1.6	2.9 ± 1.9	0.001**	−0.58
Brief Fatigue Inventory: Fatigue at its worst	Lower is better	6.8 ± 1.9	5.2 ± 2.6	<0.001	−0.69
Symptom inventory: Fatigue	Lower is better	5.8 ± 2.4	4.4 ± 2.3	0.002**	−0.62
Symptom inventory: Sleep problems	Lower is better	4.7 ± 2.9	4.2 ± 3.0	0.374	−0.18
Symptom inventory: Drowsiness	Lower is better	5.2 ± 2.5	3.0 ± 2.3	<0.001**	−0.89
Symptom inventory: Interference of symptoms with quality of life	Lower is better	4.0 ± 2.9	2.3 ± 2.6	0.001**	−0.62

* p-value derived from a two-sided, paired t-test between baseline and Day 14

** p<0.01

† Trial outcome index (fatigue) = physical + functional + fatigue subscales

‡ Functional Assessment of Cancer Therapy (FACT)-General = physical + social + emotional + functional subscales; a common measure of quality of life
